# Perfluorohexane Sulfonic Acid Disrupts the Immune Microenvironment for Spermatogenesis by Damaging the Structure of the Blood‐Testis Barrier in Mice

**DOI:** 10.1002/advs.202409383

**Published:** 2025-01-17

**Authors:** Yan Zhang, Mingxue Shu, Shilin Shan, Huiying Liu, Yucheng Zhang, Chenyang Song, Qiaoqiao Xu, Yun Fan, Chuncheng Lu

**Affiliations:** ^1^ State Key Laboratory of Reproductive Medicine and Offspring Health, Key Laboratory of Modern Toxicology of Ministry of Education, School of Public Health Nanjing Medical University Nanjing 211166 China; ^2^ Department of Epidemiology, School of Public Health Nantong University Nantong 226001 China

**Keywords:** blood‐testis barrier, mitochondria, PFHxS, sperm, testicular immunoprivileged sites

## Abstract

Perfluorohexane sulfonic acid (PFHxS) is extensively used in waterproof coatings and fire‐fighting foams, and several studies have found it to be a potential health hazard, but there is still unknown about its effects on spermatogenesis. Our results showed that PFHxS‐treated mice have significant reproductive toxicity, including a decrease in sperm count and motility, and the levels of sex hormones (*P* < 0.05). Concurrently, structural abnormalities are observed in sperm, affecting ≈60–75% of those in the PFHxS‐treated group. Additionally, it is found that the structure of the blood‐testis barrier (BTB) is damaged after PFHxS treatment, leading to higher expression levels of inflammatory cytokines in the microenvironment for spermatogenesis. Moreover, the expression of proteins associated with mitochondrial biogenesis, including PTEN‐induced kinase 1 (PINK1) and NADPH oxidase 4 (NOX4), is dysregulated in the testes after PFHxS treatment. Based on metabolome data, the differential metabolite 3‐hydroxybutanoic acid is identified in the PFHxS‐treated group, which can regulate the histone Kac levels, especially H3K4ac and H3K9ac. In summary, the results of this study suggest that in the testes of PFHxS‐treated mice, inflammatory factors disrupt the mitochondrial function and metabolic profiles and hinder the progress of gene transcription through histone Kac, ultimately causing sperm dysfunction.

## Graphical abstract

Exposure to PFHxS disrupts the immune‐isolated microenvironment and mitochondrial function homeostasis during spermatogenesis. Subsequently, it up‐regulates the metabolite β‐hydroxybutyrate which is involved in the post‐translational modification of proteins, and resulting in a decrease in histone acetylation level. Ultimately, this interference affects the transcriptional activity of genes, leading to abnormal sperm function.

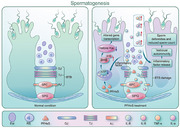

## 1 Introduction

Per‐ and polyfluoroalkyl substances (PFAS) are a variety of synthetic fluorine‐containing compounds that are widely used in industry, agriculture, and commerce, such as semiconductors, cosmetics, waterproof materials, nonstick cookware, and water‐repellent textiles.^[^ ^1^ ^]^ Most PFAS are extremely difficult to degrade in the environment owing to the highly robust perfluoroalkyl carbon portion of their structure, and some of them are bioaccumulative and widespread in the environment, wildlife, and humans.^[^ ^2^ ^]^ In recent decades, many studies have identified that exposure to long‐chain perfluoroalkyl acids (PFAAs), including perfluorooctanoic acid (PFOA) and perfluorooctane sulfonic acid (PFOS), is associated with multiple health hazards (for instance, liver injury, obesity, and hypertension).^[^ ^3^ ^]^ Considering the high toxicity of PFAAs, short‐chain PFAS with lower bioaccumulation are widely used to replace the PFAAs in some fields.^[^ ^4^ ^]^ For instance, perfluorohexane sulfonic acid (PFHxS), a short‐chain analog, has supplanted the large demand for PFOS in firefighting and electroplating.^[^ ^4^, ^5^ ^]^ As a result, more emerging PFAS alternatives, including 6:2 chlorinated polyfluoroalkyl ether sulfonate and PFHxS, are being widely detected in drinking water and human serum.^[^ ^5^, ^6^ ^]^ Moreover, another study found that some short‐chain PFAS are more mobile, persistent, and ubiquitous in surface water than PFAAs,^[^ ^7^ ^]^ suggesting that it is also potentially toxic.

PFHxS, a category of short‐chain PFAS, has been found to have an average half‐life of ≈5.3 years.^[^ ^8^ ^]^ Recently, several studies have revealed that PFHxS is significantly higher in aquatic environments, where concentrations range from 9 to 2.7 µg L^−1^, with the highest concentration of up to 7 ng L^−1^ in drinking water.^[^ ^9^ ^]^ Moreover, PFHxS has been detected in indoor sediment and dust, with a maximum concentration of up to 120 ng g^−1^.^[^ ^10^ ^]^ Furthermore, several investigations have demonstrated that exposure to PFHxS is related to a large number of diseases, including preeclampsia, polycystic ovarian syndrome, fatty liver disease, and semen quality.^[^ ^11^ ^]^ Multiple animal model studies have confirmed that PFHxS can cause neurotoxicity, developmental toxicities, metabolic syndrome, and endocrine disruption, suggesting that PFHxS is a PFAS component with potential health hazards.^[^ ^9^, ^12^ ^]^ However, there is a lack of biological data from animal studies to demonstrate that PFHxS exposure is an important cause of reproductive impairment in males. In addition, semen parameters (e.g., sperm concentration, motility, and count) exhibited inconsistent correlations with serum PFHxS levels across studies. For example, several studies involving general populations showed that exposure to PFHxS was related to declined semen quality,^[^ ^11^, ^13^ ^]^ others did not find any association with semen quality.^[^ ^13^, ^14^ ^]^ One study even reported PFHxS positively associated with sperm concentration.^[^ ^15^ ^]^ Consequently, it is imperative to conduct comprehensive and in‐depth investigations in mouse models to elucidate the relationship and underlying mechanisms between PFHxS exposure and male reproductive toxicity.

The blood‐testis barrier (BTB) is generated by neighboring Sertoli cells, which form a specific immune microenvironment for spermatogenesis known as a testicular immune privilege by tight junction (TJ) proteins, gap linkage proteins, and adherent junction (AJ) proteins^[^ ^16^, ^17^ ^]^ Under physiological conditions, low levels of proinflammatory factors are critical for spermatogenesis because they mediate spermatocyte from the base of the seminiferous tubules to the chamber.^[^ ^18^ ^]^ However, pathological inflammation response conditions, activate numerous proinflammatory factors, including macrophage chemoattractant protein 1, interleukin‐6 (IL‐6), interleukin 1β (IL‐1β), and tumor necrosis factor α (TNF‐α).^[^ ^19^ ^]^ High levels of proinflammatory factors are a major cause of abnormal spermatogenesis. For example, increased TNF‐α levels can inhibit testosterone production in Leydig cells, leading to male germ cell apoptosis and infertility.^[^ ^20^ ^]^ Several studies have identified that exposure to certain chemicals, such as polycyclic aromatic hydrocarbons or 2,2′,4,4′‐Tetrabromodiphenyl ether, can disrupt the integrity of the BTB.^[^ ^21^ ^]^ This suggests that the BTB may be a dominant target of toxicants. However, whether PFHxS impairs spermatogenesis by disrupting BTB integrity remains to be investigated.

In this study, we utilized PFHxS‐treated male mice to investigate the reproductive toxicity of PFHxS. PFHxS‐treated male mice showed reproductive toxicity similar to PFOA or PFOS exposure, such as reduced sperm counts, decreased sperm motility, and damaged the seminiferous tubules.^[^ ^3^, ^22^ ^]^ The results also revealed that PFHxS‐treated mice with an imbalanced testicular immune microenvironment. Moreover, the study identifies a metabolite 3‐hydroxybutanoic acid in PFHxS‐treated group, which has been reported to regulate histone lysine acetylation (Kac), lysine beta‐hydroxybutyrylation (Kbhb), and lysine methylation (Kme).^[^ ^23^ ^]^ Subsequent analysis revealed that the levels of histone Kac were decreased in mouse testes after PFHxS treatment, particularly histone 3 lysine 9 acetylation (H3K9ac) and histone 3 lysine 4 acetylation (H3K4ac) levels, which are strongly correlated with gene transcriptional activity.^[^ ^24^ ^]^ Our study reveals a novel mechanism underlying spermatogenic damage in male mice treated with PFHxS.

## 2 Results

### 2.1 Exposure to PFHxS Causes Male Reproductive Disorders in Mice

**Figure 1:**
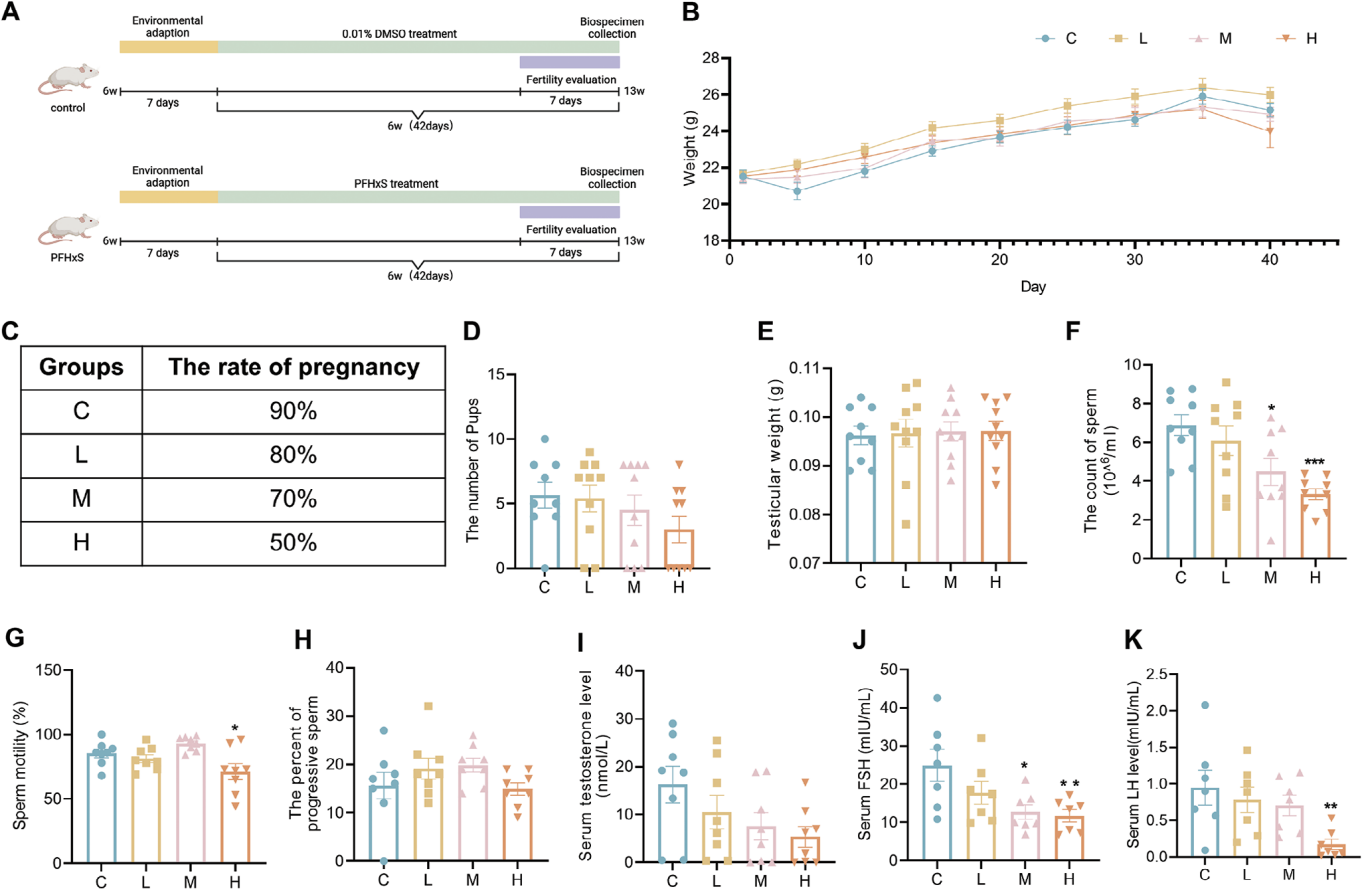
Study design and evaluation of reproductive function in PFHxS‐exposed male mice. A) Schematic map representing the research design of reproductive toxicity of PFHxS exposure. B) Body weight of mice after PFHxS exposure (n = 10). C,D) Pregnancy rate (C) and litter size (D) in PFHxS‐exposed male mice (n = 9–10). E) Testicular weight of the control and PFHxS‐exposed male mice (n = 9–10). F–H) Sperm counts (F), motility (G), and progressivity (H) were detected in the control and PFHxS‐exposed male mice (n = 8–9). I–K) Levels of T (I), FSH (J), and LH (K) (n = 7). ^*^ *P* < 0.05, ^**^ *P* < 0.01, ^***^ *P* < 0.001, and ^****^ *P* < 0.0001 represent a statistically significant difference by one‐way analysis.

To elucidate the toxicity of PFHxS on male reproduction, a mouse PFHxS exposure model was established (**Figure**  **1**A). The body weight of the mice did not alter in the different groups during the entire exposure cycle (Figure  1B). After mating with wild‐type (WT) female mice, it was found that the H group male mice had a lower conception rate (≈50%) and litter size than the C group (Figure  1C,D). The testicular weight remained constant in L, M, and H groups than that of the C group (Figure  1E), but the sperm count from the cauda epididymidis was notably lower in the M and H groups in contrast to that in the C group (Figure  1F). Moreover, it was found that sperm motility was markedly decreased in the H group compared with that in the C group (Figure  1G), but sperm progressive motility and other semen parameters (e.g., average path velocity, VAP; straight line velocity, VSL; curvilinear velocity, VCL; amplitude of lateral head, ALH) remained unchanged (Figure  1H; Figure S1A–G, Supporting Information). Meanwhile, the levels of luteinizing hormone (LH) and follicle‐stimulating hormone (FSH) were notably lower in the H group than in the C group, and the FSH level was also significantly reduced in the M group than in the C group (Figure  1I–K).

### 2.2 PFHxS Impairs Sperm Structure and Energy Metabolism

**Figure 2:**
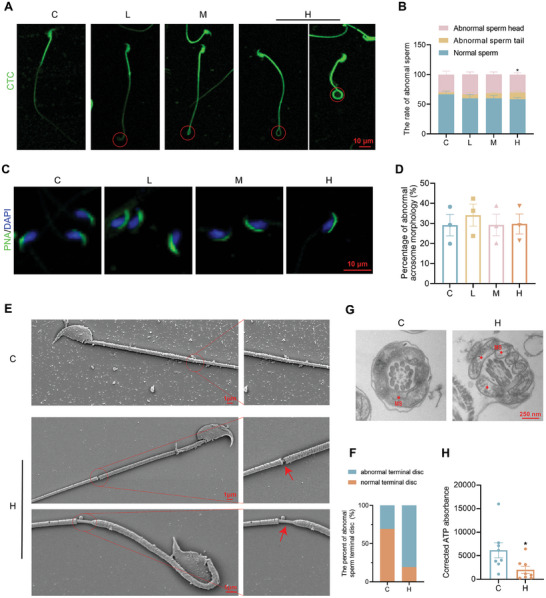
Morphological analysis of spermatozoa. A,B) Morphology of the cauda epididymal spermatozoa from the PFHxS‐exposed and control mice. Scale bar, 10 µm (n = 3). C,D) Morphometric analysis of sperm acrosomes in the cauda epididymis by fluorescein isothiocyanate‐conjugated peanut agglutinin (FITC‐PNA) staining. Nuclei were stained with DAPI (blue) and sperm acrosomes were stained with PNA (green). Scale bar, 10 µm (n = 3). E,F) Abnormalities in sperm morphology observed by SEM (n = 3). G) Ultrastructural analysis of cross‐sections of spermatozoa from the cauda epididymis obtained from the PFHxS‐exposed and control mice; red asterisks represent mitochondrial sheaths (MS) (n = 3). H) Assessment of ATP levels in the spermatozoa of the cauda epididymis (n = 8). ^*^ *P* < 0.05, ^**^ *P* < 0.01, ^***^ *P* < 0.001, and ^****^ *P* < 0.0001 represent a statistically significant difference by one‐way analysis (B and D) or unpaired *t*‐test (H).

Abnormal sperm morphology, including multiple morphological abnormalities of the flagella, head‐neck defects, and fibrous sheath dysplasia, are important causes of sperm dysfunction and male infertility.^[^ ^25^ ^]^ The morphology of sperm in the cauda epididymidis was first analyzed by immunofluorescence. The results indicated that the abnormal morphology rate of the sperm tail was significantly elevated in the H group than that of the C group (**Figure**  **2**A,B). Meanwhile, the acrosome morphology of sperm from the cauda epididymis in PFHxS‐treated mice was also examined, but no abnormalities were found when compared with the C group (Figure  2C,D). However, ≈ 75% sperms from the PFHxS‐treated mice structural defects in their annulus, as evidenced by the thinning of the region in the H group than the C group (red arrow) (Figure  2E,F). Transmission Electron Microscopy (TEM) results found that the sperm presented a normal “9 + 2” microtubule structure between H and C groups, but ≈60% of the sperm mitochondrial sheath presented conspicuous structural abnormalities in the H group than in the C group (red asterisk) (Figure  2G). Sperm are highly mobile, and to maintain continuous motion, they need to produce large amounts of adenosine triphosphate (ATP) via mitochondrial sheath oxidative phosphorylation and anaerobic glycolysis.^[^ ^26^ ^]^ Subsequently, ATP production of the sperm from the cauda epididymal was assessed in H and C groups, the results showed that the epididymal cauda sperm of H group presented much lower ATP production than those of the C group (Figure  2H). Therefore, these results demonstrated that PFHxS exposure leads to abnormalities in the structure of sperm mitochondrial sheaths, resulting in impaired ATP synthesis, and ultimately reduced sperm motility.

### 2.3 PFHxS Exposure Impairs the Structures of Testes and Spermatogenesis in Mice

**Figure 3:**
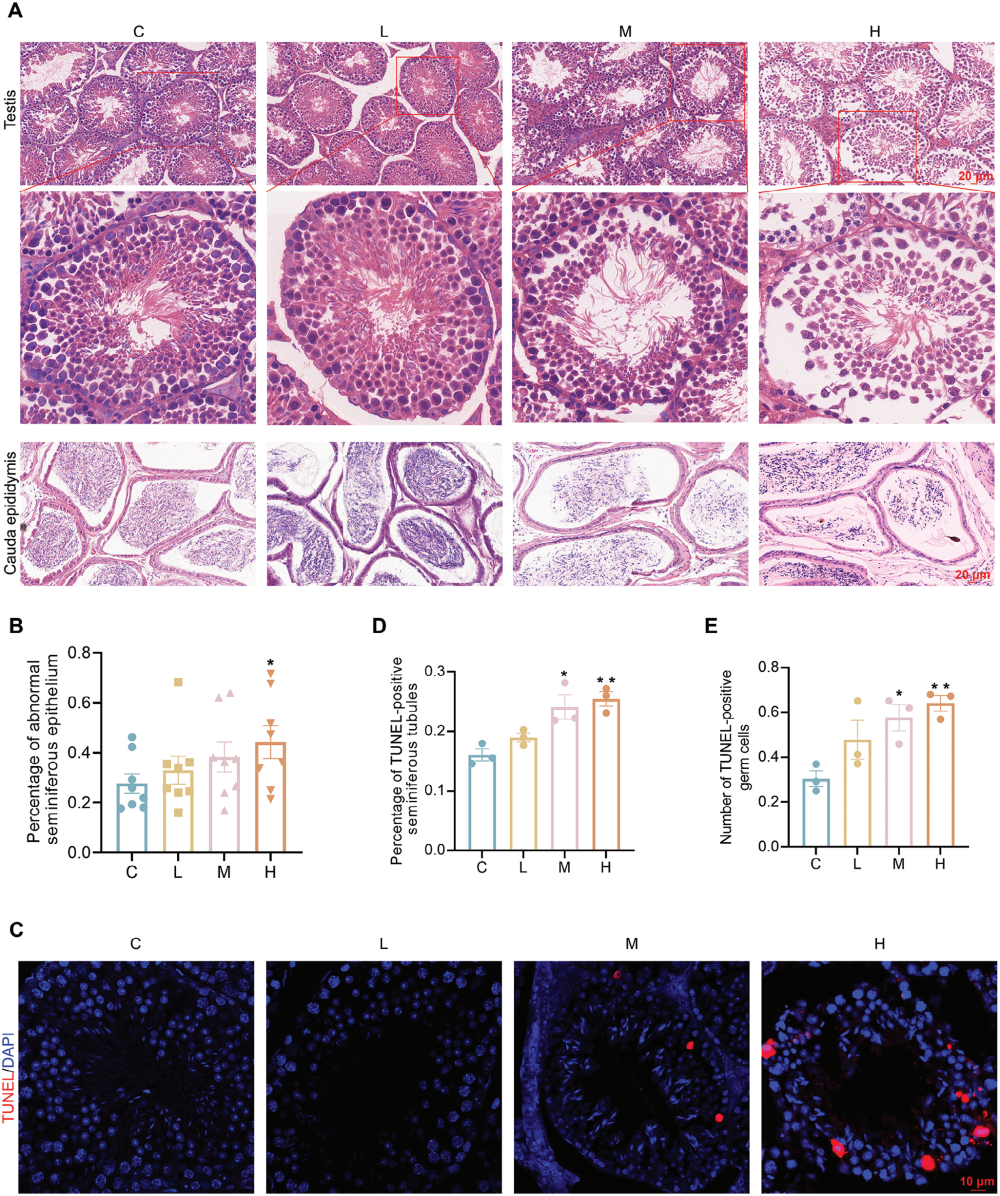
PFHxS induces testicular damage in the mouse testis. A,B) H&E staining indicates that the seminiferous epithelium was detached in the H group than that of the C group, and the sperm concentration was decreased in the cauda of the epididymis (n = 8). C–E) TUNEL staining of testes sections from the control and PFHxS‐exposed mice. Red: TUNEL‐positive cells (C), and number of TUNEL‐positive seminiferous tubules (D) and TUNEL‐positive cells per tube (E). Scale bar, 10 µm (n = 3). ^*^ *P* < 0.05 and ^**^ *P* < 0.01 represent a statistically significant difference by one‐way analysis (B,D, and E).

To assess whether PFHxS exposure affected the seminiferous epithelium structure of the testicular, histological pathological analysis of the testes was performed using H&E staining. The results showed that the seminiferous epithelium was detached from the basement membrane of seminiferous tubules in the H group compared with the C group, and the sperm concentration in the epididymis cauda was significantly reduced (**Figure**  **3**A,B). TUNEL staining was used to detect the apoptotic signals of germ cells in testicular tissue, and a notable increase was observed in both the number of TUNEL‐positive germ cells per seminiferous tubule and the percentage of TUNEL‐positive seminiferous tubules in M and H groups compared with the C group (Figure  3C–E).

### 2.4 PFHxS Exposure Damages BTB Structure and Disrupts the Expression of Intercellular Junctional Proteins

**Figure 4:**
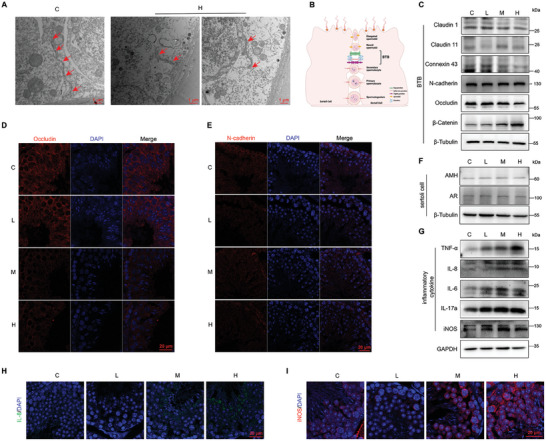
PFHxS‐induces disruption of the BTB and immune isolation microenvironment of testes. A) TEM of the testes, red arrows represent the BTB (n = 3). B) A schematic diagram showing the cellular junctions of BTB. C) The expression levels of protein of AJ, TJ, and GJ in testes (n = 3). D,E) Immunofluorescence staining of testicular sections from control and PFHxS‐exposed mice with antibodies against (D) occludin (red, TJ protein) and N‐cadherin (red, AJ protein). Scale bar, 20 µm (n = 3). F,G) Levels of Sertoli cell‐related proteins (F) and inflammatory cytokine proteins (G) (n = 3). H,I) Expression and position of IL‐8 (H) and iNOS (I) proteins in the lumen of the seminiferous tubules. Scale bar, 20 µm (n = 3).

The BTB formed by Sertoli cells is essential for sustaining normal spermatogenesis.^[^ ^27^ ^]^ TEM was performed to examine the ultrastructure of BTB. Interestingly, disintegration, vesicles, and fragmentation at the BTB were observed in the H group, while images of the C group exhibited typical BTB features (**Figure**  **4**A). BTB was established by several cellular junctions (AJ, TJ, and GJ) composed of various structural proteins (Figure  4B). To further evaluate the dysfunction of BTB, BTB‐related protein expression was assessed in different groups by western blotting, and results found the expression levels of Occludin (TJ protein), β‐Catenin (AJ protein), and Connexin 43 (GJ protein) were dysregulated in the H group compared to the C group (Figure  4C). Furthermore, the localization of several key junctional proteins was analyzed, and the results revealed that the localization of Occludin, ZO‐1 (TJ protein), and N‐cadherin (AJ protein) was not abnormal during spermatogenesis (Figure  4D,E; Figure S2A, Supporting Information). However, it was observed that PFHxS treatment did not affect the expression of AMH and AR, both Sertoli cell‐related proteins (Figure  4F). Meanwhile, the results identified that SOX9 expression remained unchanged, but the number of SOX9‐positive Sertoli cells decreased in the H group compared with the C group (Figure S2B,C, Supporting Information). Interestingly, after PFHxS exposure the levels of transcription factor GATA‐1, a protein related to the steroidogenic and germ cell differentiation,^[^ ^28^ ^]^ was reduced in Sertoli cells of seminiferous tubules at various phases during spermatogenesis (Figure S2D,E, Supporting Information). In summary, these results suggested that PFHxS treatment damages the structural integrity and homeostasis of BTB by disrupting AJ, GJ, and TJ, which may be associated with aberrant Sertoli cell function.

### 2.5 PFHxS Exposure Disrupts the Immune Isolation Microenvironment by Damaging the BTB in Mice

BTB provides an immunologically isolated microenvironment for spermatogenesis and protects germ cells from harmful effects.^[^ ^27a^ ^]^ Previous research has found that impaired TJ can cause orchitis.^[^ ^20^ ^]^ This study examined the expression of inflammatory cytokines to investigate whether the immune isolation microenvironment of the testes was disrupted after PFHxS treatment. Interestingly, IL‐8, IL‐17a, TNFα, and iNOS were notably elevated, whereas the expression of IL‐6 was not significantly altered under PFHxS treatment (Figure  4G). Moreover, immunofluorescence showed that several inflammatory cytokines infiltrated into the lumen of the seminiferous tubules (Figure  4H,I), suggesting that PFHxS exposure disrupts the immune isolation microenvironment of the testes, leading to the infiltration of inflammatory factors into the spermatogenic microenvironment in mice. Previous research has confirmed that IL‐22, IL‐17a, and IL‐24 can increase the production of reactive oxygen species (ROS), and IL‐8 can enhance oxidative metabolism and ROS generation, leading to oxidative stress.^[^ ^29^ ^]^ Therefore, malondialdehyde (MDA), superoxide dismutase (SOD), catalase (CAT), and total glutathione (GSH) levels were measured in mice testes. However, no notable differences were observed between the C and PFHxS treated groups (Figure S3A–D, Supporting Information). These findings indicated that the disruption of BTB damaged the immunologically isolated microenvironment for spermatogenesis after PFHxS treatment in mice.

### 2.6 Transcriptomic Analysis of Testes in the PFHxS Exposed and the Control Groups

**Figure 5:**
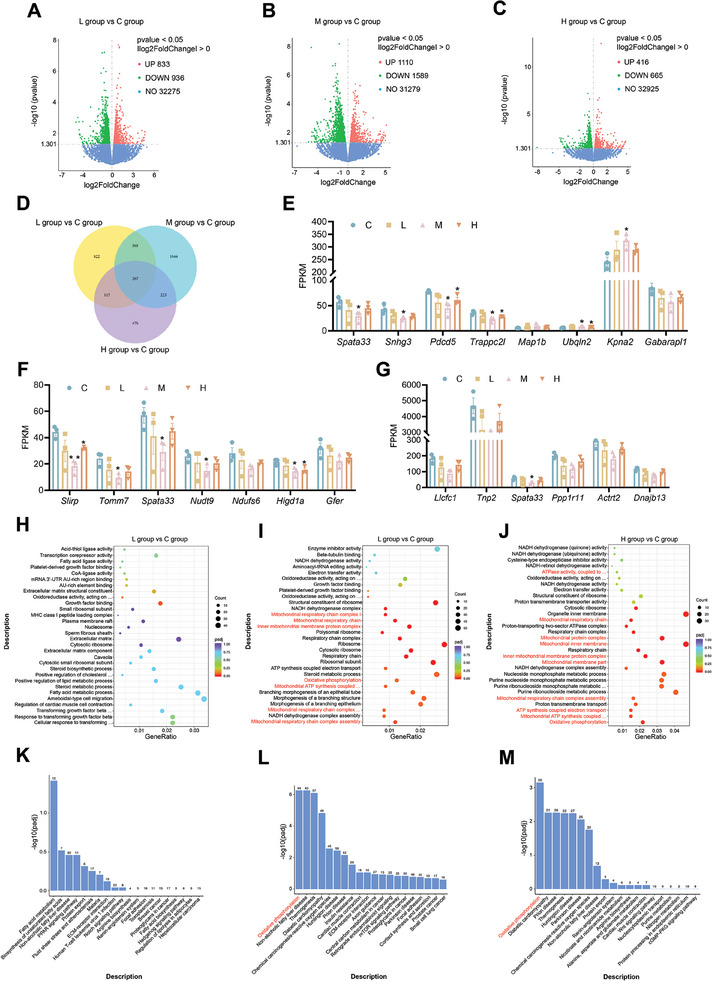
Enrichment analysis of DEGs in mice testes after PFHxS exposure. A–C) Transcriptomic analyses of the entire testis after PFHxS exposure (n = 3). D) Venn diagram demonstrating the overlap of DEGs in the three compared sets. E–G) Gene expression of autophagy‐regulated (E), mitochondrion‐regulated (F), and spermatogenesis‐regulated (G) by RNA‐seq data from testes in the PFHxS exposed and control mice (n = 3). H–J) GO analyses of DEGs in L (H), M (I), and H (J) groups (n = 3). K–M) KEGG analysis of DEGs in L (K), M (L), and H (M) groups (n = 3).

RNA‐seq was performed to analyze the global change in mouse testes genes between C, L, M, and H groups. A total of 1769, 2699, and 1081 DEGs were identified in L, M, and H groups compared with the C group (*P* < 0.05, Foldchange > 1), respectively (**Figure**  **5**A–C; Tables S2–S4, Supporting Information). The Venn diagram identified that 267 genes were differentially expressed between the three comparison groups (the L group versus C group, the M group versus C group, the H group versus C group), and 223 genes were specifically differential expressed between M versus C and H versus C comparison groups (Figure  5D). Given that the phenotypes appeared mainly in M and H groups, the functions of these 223 genes were analyzed. The results revealed that many of the 223 DGEs are closely linked to the mitochondrial function (for instance, *Slirp*, *Tomm7*), autophagy (for instance, *Snhg3*, *Trappc2l*), and spermatogenesis (for instance, *Spata33*, *Dnajb13*) (Figure  5E–G). GO analysis revealed that PFHxS treatment primarily influences biological events such as metabolism, oxidative phosphorylation, ATP synthesis, and mitochondrial function in M and H groups compared to the C group. However, no significantly different pathways were found in the L group (Figure  5H–J). Moreover, KEGG pathway enrichment analysis was conducted on the screened DEGs, and it was found that the main DEGs were enriched in the oxidative phosphorylation, ROS production, and thermogenesis pathways in M and H groups than the C group (Figure  5K–M). In conclusion, these results suggest that PFHxS may affect ATP synthesis by oxidative phosphorylation in the mitochondrion during spermatogenesis.

### 2.7 PFHxS Exposure Affects Mitochondrial Function

**Figure 6:**
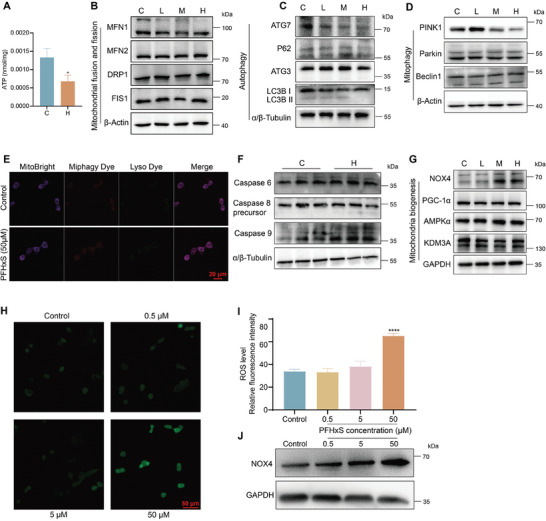
PFHxS‐induced mitophagy and mitochondria biogenesis dysfunction during spermatogenesis. A) Assay of ATP levels in the testes (n = 3). B–D) Western blotting results of selected proteins in mice testes after PFHxS treatment (n = 3), B) mitochondrial fusion and fission, (C) autophagy, (D) mitophagy. E) Representative images of mitophagy in GC‐2spd(ts) cells treated with PFHxS. The co‐localization of MitoBright, miphagy dye, and lysosome dye showing mitophagy flux in GC‐2spd(ts) cells exposed to 50 µm PFHxS for 12 h (n = 3). F,G) Western blot analysis of apoptosis pathway (F) and mitochondrial biogenesis (G) related proteins expression (n = 3). H,I) ROS detection and quantitative analysis in vitro (n = 3). J) Western blot analysis of NOX4 expression at the cellular level (n = 3). ^*^ *P* < 0.05, ^**^ *P* < 0.01, ^***^ *P* < 0.001, and ^****^ *P* < 0.0001 represent a statistically significant difference by unpaired *t*‐test (A) or one‐way analysis (I).

Notably, DEGs were commonly enriched in oxidative phosphorylation, ATP synthesis, and mitochondrial function according to GO and KEGG analyses. Accordingly, in this study, the ATP content was measured in the testes of mice, in H and C groups. The results found significantly decreased ATP production in the mouse testes of the H group than that of the C group (**Figure**  **6**A). However, it was also observed that PFHxS exposure did not affect the mitochondrial fusion and fission in mice testes by evaluating the protein expressions of MFN1, DRP1, MFN2, and FIS1 (Figure  6B). Environmental or cellular stresses stimulate autophagy by forming autophagosomes, which eliminate intracellular pathogens, protein aggregates, and damaged organelles.^[^ ^30^ ^]^ Functional autophagy can limit the activation of the inflammasome and other inflammatory responses.^[^ ^31^ ^]^ The expression of autophagy‐related proteins was also detected using western blotting in the PFHxS‐treated and control groups. The results revealed that the expression level of autophagy‐related proteins ATG7, LC3B, and a mitophagy‐related protein PINK1 were significantly reduced in M and H groups (Figure  6C,D). Subsequent analysis of autophagy flux revealed that autophagy was not induced following PFHxS treatment (Figure  6E), which may be associated with the suppression of the expression of autophagy‐related proteins after PFHxS treatment. Furthermore, caspases can digest several autophagy proteins, leading to inactivation of the autophagy program.^[^ ^32^ ^]^ The results from this study found that Caspase 9 expression was significantly elevated in group H compared with group C, with no significant changes observed in the expression of other caspase proteins (Figure  6F). This suggests that the decreased expression of autophagy‐related proteins may be related to Caspase 9 activation. Moreover, the expression of NOX4 was significantly elevated in M and H groups (Figure  6G). Previous study has found that NOX4 upregulation promotes mitochondrial ROS (mtROS) production, mitochondrial dysfunction, and apoptosis.^[^ ^33^ ^]^ Subsequently, the results showed that ROS levels were significantly elevated in the PFHxS‐treated group at a concentration of 50 µM (Figure  6H,I), accompanied by increased NOX4 expression in GC‐2spd(ts) cells (Figure  6J). Consequently, these results indicate that high‐dose of PFHxS treatment resulted in impaired mitochondrial function and induced excessive ROS production via the NOX4 pathway, and triggered apoptosis by inhibiting the activation of cellular autophagy.

### 2.8 PFHxS Disturbs the Metabolic Profiles of Testes

**Figure 7:**
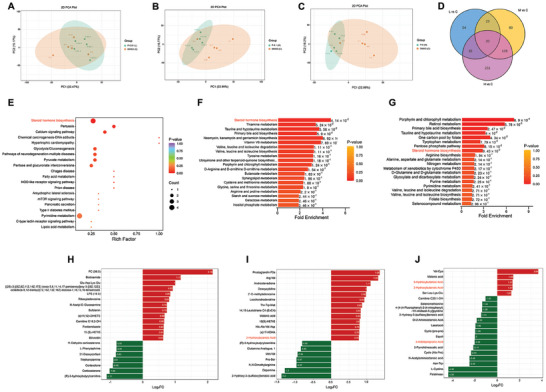
Metabolomic analysis of testes in the PFHxS exposure and control mice. A–C) PCA of metabolomic data in L (A), M (B), and H (C) groups (n = 5). D) Venn diagram demonstrating the shared differential metabolites in three compared sets (n = 5). E–G) KEGG enrichment of differential metabolites in 128 differential metabolites were exclusively detected in M and H groups (E), the differential metabolites from M (F) and H groups (G) (n = 5). H–J) Top 20 differential metabolites in L (H), M (I), and H (J) groups (n = 5).

Several studies have suggested that mitochondrial dysfunction and oxidative stress play an important role in the pathogenesis of metabolic diseases or disorders.^[^ ^34^ ^]^ The changes in testicular metabolite profiles may further indicate some molecular pathways involved in the spermatogenesis response to PFHxS exposure. Accordingly, a metabolomic analysis of testicular tissues from PFHxS‐treated mice was performed. Principal components analysis (PCA) analysis showed that the PFHxS‐treated M and H groups were different from the C group, while the L group had high concordance with the C group (**Figure**  **7**A–C). After PFHxS treatment, the heatmap revealed 112 differential metabolites, including 10 down‐regulated and 102 up‐regulated metabolites in the L group (Figure S4A; Table S5, Supporting Information). For the M group, the results identified 251 differential metabolites, including 26 down‐regulated and 225 up‐regulated metabolites (Figure S4B; Table S6, Supporting Information). Moreover, 283 up‐regulated and 111 down‐regulated metabolites were detected in the H group (Figure S4C; Table S7, Supporting Information). The Venn diagram illustrated that 128 differential metabolites were exclusively detected in M and H groups (apparent reproductive abnormality phenotypes were observed in both groups after PFHxS exposure) (Figure  7D). To determine the function of these 128 differential metabolites, KEGG functional annotation was performed, and the results showed that the most enriched pathways included steroid hormone biosynthesis, pertussis, and calcium signaling pathways (Figure  7E). Subsequently, the differential metabolites from M and H groups were analyzed, and it was evident that the pathways of steroid hormone biosynthesis were found in both groups (Figure  7F,G). K‐Means cluster analysis showed that 385 metabolites exhibited dose‐dependent changes, including 264 with an increasing trend and 121 with a decreasing trend (Figure S4D–I, Supporting Information). KEGG analysis was performed to identify the key pathway related to male reproduction based on K‐Means cluster analysis, and the steroid hormone biosynthesis pathway was found in subclass five (Figure S5A–D, Supporting Information), suggesting that PFHxS exposure significantly alters the metabolic pathway of sex hormone synthesis.

### 2.9 PFHxS Induces Histone Lysine Acetylation (Kac) Alterations During Spermatogenesis

**Figure 8:**
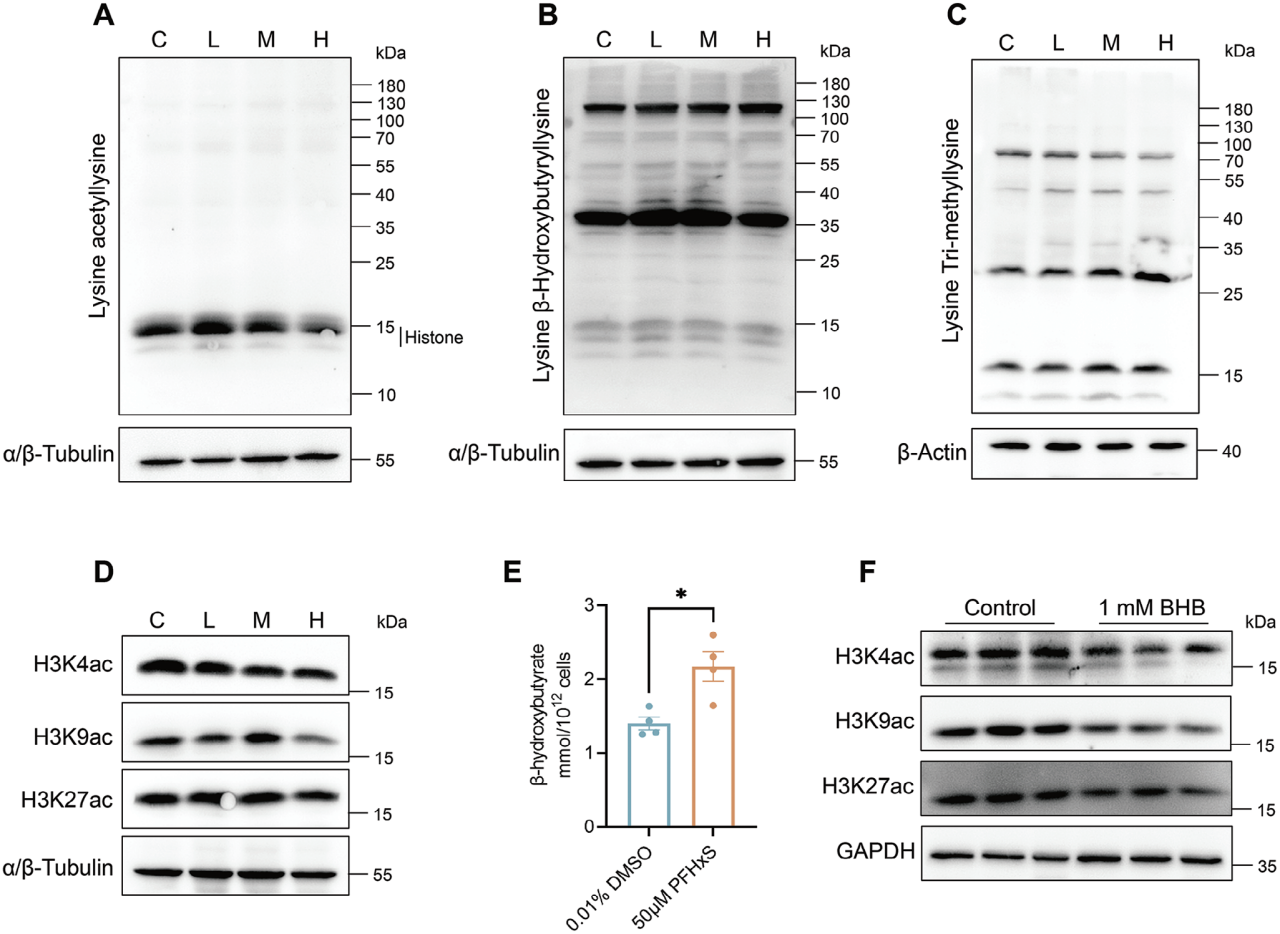
Western blotting analysis of the level of protein post‐translational modification expression in mouse testis. A) Lysine acetylation (n = 3). B) β‐Hydroxybutyryllysine (n = 3). C) Tri‐methyllysine (n = 3). D) Expression of H3K9ac, H3K4ac, and H3K27ac in mouse testis (n = 3). E) Quantitative analysis of the BHB levels following PFHxS exposure in GC‐2spd(ts) cells (n = 4). F) Expression of H3K9ac, H3K4ac, and H3K27ac in GC‐2spd(ts) cells (n = 3).

Figure  7H–J shows the top 20 differential metabolites identified in the PFHxS exposure group, including 3‐hydroxybutanoic acid, 2‐hydroxybutanoic acid, 2′‐O‐methyladenosine, and 3‐indolepropionic acid, which were only detected in M and H groups. Then, the levels of Kac, Kbhb and Kme were investigated in testes by western blotting, and the results indicated that the histone Kac level was decreased in the H group compared with the C group, while the levels of Kbhb and Kme were not altered in the PFHxS treated groups (**Figure**  **8**A,C). We further analysis the levels of histone acetylation, and the results showed that the levels of H3K9ac and H3K4ac were obviously reduced in the H group than that in the C group (Figure  8D). Global histone hyperacetylation has been reported to be necessary for spermiogenesis, such as H3K9ac is essentially related to transcriptional activation in human cell, and chromatin remodeling during spermiogenesis in mammalian,^[^ ^35^ ^]^ suggesting that PFHxS exposure alters gene transcriptional profiles by decreasing H3 acetylation levels during spermatogenesis.

To confirm that PFHxS is involved in the regulation of acetylation through 3‐hydroxybutanoic acid, known as β‐hydroxybutyrate (BHB), PFHxS or BHB treatment GC‐2spd(ts) cells were used in the study. The level of BHB was evaluated in the PFHxS‐treated group compared with control group in vitro (Figure  8E), which consistent with the results of in vivo. Subsequently, we assessed the changes in histone acetylation levels in vitro, and found that H3K4ac, H3K9ac and H3K27ac levels were notably reduced after BHB treatment at 1 mM concentration in the GC‐2spd(ts) cells (Figure  8F), suggesting that PFHxS is involved in regulating the levels of H3 acetylation by BHB.

## 3 Discussion

Reduced fertility or infertility can result from lifestyle factors (obesity and tobacco), testicular dysfunction, aging, congenital anatomical factors, gonadotoxic exposures, and adverse environmental factors.^[^ ^36^ ^]^ Several recent studies suggest that the quality and quantity of sperm in some patients can be reduced irreversibly due to adverse environmental factors, such as endocrine‐disrupting chemicals, drugs, and toxicants, rather than genetic factors.^[^ ^37^ ^]^ PFHxS, a common PFOS alternative, has many applications, including water‐proof coatings, fire‐fighting foams, and surfactants.^[^ ^8b^ ^]^ Several recent studies have indicated that the average concentrations of PFHxS in the general population serum range from 0.04 to 48.43 ng mL^−1^, which is comparable to the PFOS exposure levels in the general population serum (concentration: 10–80 ng mL^−1^).^[^ ^38^ ^]^ Although several studies have indicated that it exhibits strong embryo developmental toxicities and neurotoxicity,^[^ ^9^, ^39^ ^]^ the reproductive toxicity data on PFHxS are relatively limited.

The results of this study identified that exposure to PFHxS can induce male reproductive toxicity in mice at concentrations of 0.1 and 5 mg kg^−1^ day^−1^. PFHxS impairs spermatogenesis, as evidenced by reduced sperm counts, decreased sperm motility, affected sex hormone levels (testosterone, FSH, and LH), and increased sperm malformation rates. Sperm motility depends on the inner and outer axonemal dynein arms, which help the microtubule bilayer slide by consuming ATP.^[^ ^40^ ^]^ Consequently, ATP, a necessary element for sperm motility, is predominantly supplied by the mitochondrial sheath in the midpiece of the sperm flagellum.^[^ ^41^ ^]^ The sperm mitochondrial sheath presented conspicuous structural abnormalities and reduced ATP production in cauda epididymal sperm after 5 mg kg^−1^ day^−1^ PFHxS exposure. As a result, these results provide direct evidence linking PFHxS exposure to sperm dysfunction.

The testis is a special organ that serves as the primary site of germ cell development and generates a particular physiological microenvironment for spermatogenesis.^[^ ^42^ ^]^ BTB, a physical barrier and junction, is unique to the testis and is formed by Sertoli cells.^[^ ^27^, ^43^ ^]^ BTB has long been identified as having immunoprotective properties for novel proteins expressed by spermatids and spermatocytes.^[^ ^43^ ^]^ However, whether PFHxS disrupts the testicular immunoprivileged sites and causes an inflammatory response during spermatogenesis is unknown. This study demonstrated that PFHxS exposure inhibited the expression of TJ and AJ proteins, ultimately destroying the structural integrity of BTB, which subsequently causes the high level of inflammatory cytokines, such as IL‐17a, IL‐8, TNF‐α, and iNOS, to infiltrate into the lumen of the seminiferous tubules. However, the change in IL‐6 expression was not significant after PFHxS treatment, which may be related to the diverse sources and functions of cytokines. For instance, IL‐1β, IL‐6, IL‐8, IL‐12, TNF‐α are generally recognized as pro‐inflammatory cytokines, while IL‐4, IL‐6, IL‐10, IL‐11, IL‐13 are considered anti‐inflammatory cytokines. Notably, IL‐6 has the unique property of acting as both a pro‐ and anti‐inflammatory cytokine.^[^ ^44^ ^]^ The overexpression of inflammatory cytokines can damage the development of male germ cells, leading to infertility.^[^ ^20^ ^]^ Consequently, we hypothesized that PFHxS exposure triggers an immune response against spermatocytes and spermatids, eventually leading to mature sperm dysfunction.

Inflammation and infection are causative factors in male reproductive disorders that can result in subfertility or infertility, which negatively affects spermatogenesis via impaired mitochondrial activity, ROS production, and disruption of sex hormone synthesis.^[^ ^45^ ^]^ In this study, transcriptome sequencing data demonstrated that the DEGs were predominantly enriched in mitochondrial function and ATP synthesis pathways in the PFHxS exposure groups at concentrations of 0.1 and 5 mg kg^−1^ day^−1^ (two groups with significant reproductive abnormalities). Furthermore, it was discovered that the expression of the mitophagy protein PINK1 was reduced in the testes of mice exposed to PFHxS at concentrations of 0.1 and 5 mg kg^−1^ day^−1^. The function of mitochondria is not limited to energy production; they also play diverse roles in steroid hormone production, maintenance of normal flagellar structure for spermatozoa, and germ cell differentiation and death during gamete formation.^[^ ^46^ ^]^ PINK1 plays an essential role in monitoring mitochondrial integrity by phosphorylating Parkin to initiate mitophagy.^[^ ^47^ ^]^ The absence of PINK1 has been demonstrated to diminish electron transport chain (ETC) function via reduced enzymatic activity of ETC complex I and IV, altered mitophagy, increased oxidative stress, and leading cells to apoptosis in vitro and vivo.^[^ ^48^ ^]^ Moreover, PINK1‐deficient mice exhibited more severe inflammatory responses, with elevated levels of the proinflammatory cytokines *Tnfa* and *Il6*,^[^ ^49^ ^]^ consistent with our results. In another study, *Pink1*‐deficient Drosophila exhibited mitochondrial defects and male sterility.^[^ ^50^ ^]^ Interestingly, in this study, we observed a decrease in the expression of autophagy‐related proteins such as PINK1, ATG7, and LC3B following exposure to PFHxS. Subsequent investigations also failed to detect activation of autophagy in PFHxS‐treated cells, suggesting that this may be associated with the suppression of expression of autophagy‐related proteins post‐PFHxS treatment. Consequently, we hypothesized that exposure to PFHxS damages the structural integrity of BTB, which subsequently disrupts the testicular immunoprivileged sites, and the reduced level of the mitophagy‐related protein PINK1 exacerbates the inflammatory response, eventually causing sperm dysfunction.

In addition to being known as the “powerhouse” of the cell, mitochondria serve as the hub of metabolism.^[^ ^51^ ^]^ Several studies have confirmed that disruption of the testicular microenvironment and mitochondrial dysfunction are major factors triggering metabolic profile disorders in mice.^[^ ^52^ ^]^ This study revealed that the metabolic profile of the testes is significantly altered after exposure to PFHxS, suggesting that PFHxS may disrupt the normal metabolic profile by damaging the testicular BTB and microenvironment in mice. Among the differentially metabolized substances, BHB is involved in the transcriptional regulation of genes through the regulation of histone modifications including Kac, Kbhb, and Kme,^[^ ^23^ ^]^ was significantly elevated in the H group. A previous study identified that a ketogenic diet can increase BHB, which further reduces the acetylation of p53 and controls gene expression in relationship with the ketogenic diet.^[^ ^53^ ^]^ The results of this study demonstrated that histone Kac, H3K9ac, and H3K4ac levels were significantly reduced in the 5 mg kg^−1^ day^−1^ PFHxS exposure group. The level of H3K9ac is correlated with the progression of spermiogenesis,^[^ ^54^ ^]^ and it regulates the progression of gene transcription in pachytene spermatocytes or round spermatids.^[^ ^55^ ^]^ Additionally, H3K4ac, as a marker of active transcription, is related to mRNA and eRNA transcription.^[^ ^56^ ^]^ Therefore, we hypothesized that PFHxS exposure may influence histone acetylation (H3K9ac and H3K4ac) via BHB and consequently be involved in gene transcriptional regulation during spermatogenesis. However, there is still a need for more studies to confirm this speculation.

## 4 Conclusion

In conclusion, these findings revealed that PFHxS exposure damages the structural integrity of BTB, which disrupts the testicular immunoprivileged sites, subsequently leading to an inflammatory response, impairing mitochondrial activity, and eventually causing sperm dysfunction during spermatogenesis. Additionally, this study identified that PFHxS may mediate histone acetylation by the metabolite BHB, which in turn affects the transcriptional program of genes by histone Kac during spermatogenesis. The present study provides new directions and insights for follow‐up studies of abnormal spermatogenesis after exposure to PFAS. In the future, our focus will be on the molecular mechanisms of chemical‐induced testicular immune microenvironment imbalance.

## 5 Experimental Section

### Experimental Animals and Chemicals

PFHxS (CAS: 355‐46‐4, >99.0% pure) was procured from Sigma‐Aldrich, and diluted in 0.01% dimethyl sulfoxide (DMSO) (Sigma‐Aldrich, D2650) to dispense the stock solution. C57BL/6 mice aged six‐weeks were obtained from the Animal Core Facility of Nanjing Medical University (License Key: SYXK(SU) 2020–2022). The mice were adaptor fed for 10 days under specific pathogen‐free conditions before formal experimentation. Then, the mice were randomized into four groups: three PFHxS‐treated groups and one control group (C, 0.01% DMSO). For six weeks, mice in the PFHxS exposure groups were treated with PFHxS at doses of 0.01 mg kg^−1^ day^−1^ (low‐dose group, L), 0.1 mg kg^−1^ day^−1^ (medium‐dose group, M), and 5 mg kg^−1^ day^−1^ (high‐dose group, H) by oral gavage. After 42 days of treatment, the testis, epididymis and sperm were retrieved for subsequent analysis. All studies involving animals were approved by the Institutional Animal Care and Use Committee of Nanjing Medical University (IACUC‐2009005).

### Computer‐Assisted Sperm Analysis

The sperm quantity from the cauda epididymis, including sperm motility, progression, and other semen parameters like average path velocity (VAP), straight line velocity (VSL), curvilinear velocity (VCL) and mean amplitude of the lateral head displacement (ALH), were analyzed by computer‐assisted semen analysis (CASA), as described previously.^[^ ^57^ ^]^ Briefly, the isolated distal cauda epididymides were immediately transferred to 700 µL of non‐capacitated medium and incubated at 37 °C for 10 min. The quantity of sperm from the epididymal cauda was then analyzed using the LUNA‐II (Logos Biosystems, Korea).

### Histological Analysis, TUNEL Assay, and Immunofluorescence Staining

Histological analysis of the epididymis and testes has been described in previous research.^[^ ^58^ ^]^ Briefly, the testes and epididymis were fixed in 4% paraformaldehyde and then dehydrated with a gradient alcohol solution (70%, 80%, 90%, and 100% alcohol, respectively) before being embedded in paraffin. Subsequently, the testes were sliced into 4 µm sections, deparaffinized, hydrated, and immersed in hematoxylin and eosin (H&E) for 5 and 2 min, respectively. The testes sections were imaged using a Pannoramic SCAN (3D HISTECH, Hungary).

TUNEL assay was conducted using the TUNEL BrightRed Apoptosis Detection Kit (Vazyme, China) following the manufacturer's protocol. Immunofluorescence has been described in a previous study.^[^ ^58^ ^]^ Briefly, the paraffin sections were blocked in 5% bovine serum albumin (BSA) at room temperature (RT) for 2 h after being dewaxed, rehydrated, and antigen retrieval. The primary antibodies were then added to the tissue of testicular sections and incubated at 4 °C overnight. The next day, phosphate‐buffered saline (PBS) was used to wash the testicular sections, and the samples were incubated in the secondary antibodies for 2 h at RT in a darkroom. Images were captured using a LSM900 laser scanning confocal microscope (ZEISS, Germany). Antibodies information is detailed in Table S1 (Supporting Information).

### Western Blotting

Phenylmethanesulfonyl fluoride (Beyotime Biotechnology, China) and RIPA Lysis Buffer (Beyotime Biotechnology, China) were used to extract the protein from the testes. The suspensions were sonicated and kept on ice for 30 min. The supernatants were collected after centrifuging the lysates at 13,000 g for 30 min at 4 °C. Measuring the concentration of proteins using the BCA Protein Quantification Kit (Vazyme, China). The protein samples were separated on sodium dodecyl sulfate‐polyacrylamide gel electrophoresis and transferred to poly‐vinylidene fluoride (PVDF) membranes. The PVDF membranes were blocked in 5% experimental grade non‐fat milk or BSA for 1–2 h at RT and then incubated in primary antibodies overnight at 4 °C. The next day, the PVDF membranes were immediately incubated in the secondary antibodies at RT for 2 h after three times washes with Tris‐Buffered saline Tween buffer. The SuperSignal West Femto Chemiluminescent Substrate (Tanon, China) was used to visualize the signal of target proteins. Antibodies information is detailed in Table S1 (Supporting Information).

### Scanning Electron Microscopy (SEM)

Mouse epididymal tail sperm were fixed with 2.5% glutaraldehyde (diluted in PBS). The liquid was dropped onto a cell slide, and the sperm concentration was observed under a microscope. After air drying, the slides were covered with 2.5% glutaraldehyde, and were placed in a refrigerator at 4 °C overnight. After washing four times with PBS buffer, the slides were fixed for 1 h, followed by gradient dehydration treatment, critical point drying treatment, and spray gold coating of the sperm samples. Images were captured using SEM (JSM‐7900F, JEOL).

### Transmission Electron Microscopy (TEM)

Testicular tissue was treated with 2.5% glutaraldehyde overnight at 4 °C. The sperm were centrifuged at 4 °C, the supernatant was discarded, and 2.5% glutaraldehyde was slowly added overnight at 4 °C. Then, 1% osmic acid was added for further fixation for another 2–3 h. The testes and sperm were stained with 2% uranium acetate, then dehydrated using a gradient acetone method, followed by embedding and preparing of ultrathin sections for further staining. Ultrathin slice was imaged using TEM (Tecnai G2 Spirit Bio TWIN, FEI).

### Measurement of Adenosine Triphosphate (ATP) Concentration in Testis and Spermatozoa

ATP Assay Kit (Beyotime, China) was used to determine the ATP concentration according to the manufacturer's instructions. Briefly, homogenized testicular tissue and spermatozoa from the caudal epididymides were incubated in 200 µL ATP lysate. The supernatant was collected for subsequent experiments after centrifugation at 4 °C. The ATP standard solution was serially diluted to 0.01, 0.03, 0.1, 0.3, 1, 3, and 10 µm concentrations using ATP assay lysates. Then, 100 µL of the ATP detection solution was added to the detection wells and placed at RT for 5 min, followed by adding 20 µL sample or standard solution to the tubes and immediately mixed using a pipette. A luminometer was used to determine the relative light unit value.

### RNA Sequencing (RNA‐seq) Assay

RNA‐seq analysis of testicular tissue was conducted by Novogene Co. Ltd. (Tianjin, China). RNA integrity was evaluated using the RNA Nano 6000 Assay Kit with a Bioanalyzer 2100 system (Agilent Technologies, USA). The cDNA library was prepared according to the standard Illumina RNA‐seq instructions, sequenced on the Illumina Novaseq platform, and 150 bp paired‐end reads were obtained. Raw data were preprocessed using Trimmomatic software to remove low‐quality reads and adaptor contamination. HISAT2 was selected as the alignment tool to align the paired‐end clean reads with the mm10 genome, and DESeq2 software (1.20.0) was used for differential expression analysis across the two combinations. ClusterProfiler (3.8.1) software was used to perform gene ontology (GO) enrichment analysis on differentially expressed genes (DEGs) as well as their statistical enrichment in the Kyoto Encyclopedia of Genes and Genomes (KEGG) pathway.

### Determination of Intracellular ROS and β‐hydroxybutyrate

A mouse spermatocyte‐derived GC‐2spd(ts) cell line used as an in vitro cell model in this study.^[^ ^59^ ^]^ GC‐2spd(ts) cells were seeded in confocal dishes at a density of 1 × 10^5^ cells dish^−1^ and cultured in an incubator for 12 h. Then it was treated with 0.01% DMSO or various concentrations of PFHxS (0.5, 5, and 50 µm) for 12 h. These concentrations are markedly lower than the reported for the effect concentration causing 50% reduction in cell viability (325.05 µm).^[^ ^9^, ^60^ ^]^ Next, incubated cells with serum‐free medium containing 10 µm DCFH‐DA for 30 min at 37 °C. The cells were then washed twice with serum‐free medium. The detection method was carried out by referring to a reactive oxygen species (ROS) detection kit (Beyotime Biotechnology, China). Images were captured using a LSM900 laser scanning confocal microscope (ZEISS, Germany).

β‐hydroxybutyrate (BHB) level was determined by β‐Hydroxybutyrate Colorimetric Assay Kit (Elabscience, E‐BC‐K785‐M). In brief, GC‐2spd(ts) cells were treated with 0.01% DMSO or 50 µM PFHxS for 24 h. Then, collect the cells by centrifugation (centrifuged at 1000 g for 10 min at 4 °C) and measure the cell concentration. Subsequently, the cell suspension was sonicated with 20 cycles, each lasting for 1 min. Finally, centrifuge cell suspension at 1000 g for 10 min at 4 °C and remove the supernatant for measuring. The level of BHB is normalized by the number of cells in each sample.

### Mitophagy Flux Detection In Vitro

To assess mitophagy flux, GC‐2spd(ts) cells were stained with a Mitophagy Detection Kit (Dojindo, MD01). Plate GC‐2spd(ts) cells at a concentration of 1 × 10^5^ cells/dish in confocal dishes and culture them in an incubator for 12 h. After staining with Mtphagy Dye (containing 25 nM of MitoTracker Deep Red [Yeasen, China]) for 30 min at 37 °C, the cells were washed twice and treated with 0.01% DMSO or 50 µM PFHxS for 12 h. Thereafter, the cells were treated with Lyso Dye for 30 min at 37 °C and washed twice with serum‐free medium. Fluorescent images were captured using a LSM900 laser scanning confocal microscope (ZEISS, Germany).

### Untargeted Metabolomic Analysis

Testis samples (20 mg) from each group were used for metabolomic analysis, as previously described.^[^ ^61^ ^]^ Briefly, each testis sample was pretreated with 10 µL internal standard A (containing creatinine, L‐phenylalanine acid, horse uric acid, glutarate, niacin, valine N‐acetaminophenol, and thymine), 460 µL methanol, 150 µL ultrapure water, 10 µL internal standard C (containing tetracosanoic acid), and 10 µL internal standard B (containing decanoic acid). Pretreated samples were centrifuged at 20,000 g for 15 min at 4 °C to obtain the supernatant, which was then dried and resuspended. A Q‐Exactive mass spectrometer (QEMS) (Thermo Fisher Scientific, USA) and UPLC Ultimate 3000 system (Dionex, Germering, Germany) were used to identify metabolomic profiles in positive and negative modes simultaneously. The data analysis was conducted according to the previous description.^[^ ^62^ ^]^

### Statistical Analyses

All data (mean ± standard error of the mean) were analyzed using GraphPad Prism software (version 9.0; GraphPad, San Diego, CA). A one‐way analysis of variance was used for comparisons among multiple groups, and the non‐parametric t‐test was used for comparison between two groups. ^*^ *P* < 0.05, ^**^ *P* < 0.01, ^***^ *P* < 0.001, and ^****^ *P* < 0.0001 represent a statistically significant difference.

### Ethics Approval Statement

All studies involving animals were approved by the Institutional Animal Care and Use Committee of Nanjing Medical University (IACUC‐2009005)

## Conflict of Interest

The authors declare no conflict of interest.

## Author Contributions

Y.Z., M.S., and S.S. contributed equally to this study and should be regarded as joint first authors. Y.Z., M.S., and S.S. performed the experiment and wrote the manuscript; Y.Z. performed data analysis; H.L. and C.S. assisted with the experiments and provided technical help; Q.X. performed conceptualization and supervision; C.L. and Y.F. performed conceptualization, funding acquisition, and project administration.

## Supporting information



Supporting Information

## Data Availability

The data that support the findings of this study are available in the supplementary material of this article.;
