# Supplementary material for: Perfluorohexane Sulfonic Acid Disrupts the Immune Microenvironment for Spermatogenesis by Damaging the Structure of the Blood‐Testis Barrier in Mice
Source: Adv Sci (Weinh). 2025 Jan 17;12(10):2409383. doi: 10.1002/advs.202409383 (PMC11904953; doi:10.1002/advs.202409383)
Supplement: Supplementary file 1 — Supporting Information [file ADVS-12-2409383-s001.docx]

**Table S1. Primary and secondary antibodies used in this study**

| **Primary antibodies** | **Source** | **Catalog number** |
| --- | --- | --- |
| Androgen receptor Monoclonal antibody | Proteintech | 66747-1-Ig (WB: 1:1000) |
| Rabbit anti-SOX9 | Abcam | ab185230 (IF: 1:250) |
| DRP1 Rabbit Polyclonal Antibody | HUABIO | HA500487 (WB: 1:1000) |
| Rabbit polyclonal anti- Connexin 43 | Proteintech | 26980-1-AP (WB: 1:1000; IF: 1:500) |
| Rabbit polyclonal anti-occludin | Proteintech | 27260-1-AP (WB: 1:2000; IF: 1:500) |
| Rabbit polyclonal anti- beta-catenin | Proteintech | 51067-2-AP (WB: 1:5000) |
| Rabbit polyclonal anti-GAPDH | Affinity | AF7021 (WB: 1:5000) |
| Rabbit polyclonal Anti-Tubulin beta | Affinity | AF7011 (WB: 1:3000) |
| Rabbit polyclonal Anti-actin beta | Affinity | AF7021 (WB: 1:5000) |
| ZO-1 (R40.76) | Santa Cruz Biotechnology | sc-33725 (IF: 1:50) |
| Rabbit polyclonal anti-claudin-1 | Proteintech | 28674-1-AP (WB: 1:1000) |
| TNF alpha Antibody | Affinity | AF7014 (WB: 1:500) |
| IL6 Antibody | Affinity | DF6087 (WB: 1:1000) |
| IL8 Antibody | Affinity | DF6998 (WB: 1:1000; IF: 1:250) |
| iNOS Antibody | Affinity | AF0199 (WB: 1:1000; IF: 1:250) |
| IL17A Antibody | Affinity | DF6127 (WB: 1:1000) |
| MFN1 Antibody | Thermo | MA5-24789 (WB: 1:1000) |
| MFN2 Antibody | Thermo | PA5-120185 (WB: 1:1000) |
| Anti-FIS1 antibody | Thermo | PA5-22142 (WB: 1:1000) |
| Anti-PINK1 Rabbit Polyclonal Antibody | HUABIO | ER1706-27 (WB: 1:1000) |
| Parkin Recombinant Rabbit Monoclonal Antibody | HUABIO | ET1702-60 (WB: 1:1000) |

| Beclin 1 Recombinant Rabbit Monoclonal Antibody | HUABIO | HA721216 (WB:1000) |
| --- | --- | --- |
| NOX4 Recombinant Rabbit Monoclonal Antibody | HUABIO | ET1607-4 (WB:1000) |
| Claudin 11 Rabbit Polyclonal Antibody | HUABIO | ER65869 (WB:1000) |
| N-cadherin Polyclonal antibody | Proteintech | 22018-1-AP (WB:1000; IF: 1:200) |
| AMH Polyclonal antibody | Proteintech | 14461-1-AP (WB: 1:1000) |
| PGC1 alpha/beta Antibody | AiFang biological | AF301056 (WB: 1:1000) |
| GATA-1 Antibody | Santa Cruz Biotechnology | sc-266 (IF: 1:50) |
| ATG7 Polyclonal antibody | Proteintech | 10088-2-AP (WB: 1:1000) |
| LC3B Antibody | Cell Signaling Technology | 2775 (WB: 1:1000) |
| ATG3 Antibody | Affinity | AF7587 (WB: 1:1000) |
| Anti-β-Hydroxybutyryllysine Rabbit mAb | PTMBio | PTM-1201RM (WB: 1:1000) |
| Acetylated-Lysine Antibody | Cell Signaling Technology | 9441 (WB: 1:1000) |
| SQSTM1/p62 Antibody | Cell Signaling Technology | 5114 (WB: 1:1000) |
| Anti-Tri-Methyllysine Rabbit pAb | PTMBio | PTM-601 (WB: 1:1000) |
| AMPK alpha 1 Recombinant Rabbit Monoclonal Antibody | HUABIO | ET1608-40 (WB: 1:1000) |
| KDM3A / JHDM2A Recombinant Rabbit Monoclonal Antibody | HUABIO | HA721729 (WB: 1:1000) |
| Acetyl-Histone H3 (Lys9) (C5B11) Rabbit mAb | Cell Signaling Technology | 9649 (WB: 1:1000) |
| Acetyl-Histone H3 (Lys14) (D4B9) Rabbit mAb | Cell Signaling Technology | 7627 (WB: 1:1000) |
| Acetyl-Histone H3 (Lys27) (D5E4) XP® Rabbit mAb | Cell Signaling Technology | 8173 (WB: 1:1000) |
| Caspase 6/p18/p11 Polyclonal antibody | Proteintech | 10198-1-AP (WB: 1:1000) |
| Caspase 8/p43/p18 Polyclonal antibody | Proteintech | 13423-1-AP (WB: 1:1000) |
| Cleaved Caspase-9 (Asp353) Antibody | Cell Signaling Technology | 9509 (WB: 1:1000) |
| **Secondary antibodies** | **Source** | **Catalog number** |
| Alexa Fluor 488 Rabbit | Beyotime | A0423 |
| Alexa Fluor 488 Mouse | Beyotime | A0428 |
| Alexa Fluor 555 Rabbit | Beyotime | A0453 |
| Alexa Fluor 555 Mouse | Beyotime | A0460 |
| HRP-labeled Goat Anti-Mouse IgG (H+L) | Beyotime | A0216 |
| HRP-labeled Goat Anti-Rabbit IgG(H+L) | Beyotime | A0208 |

**
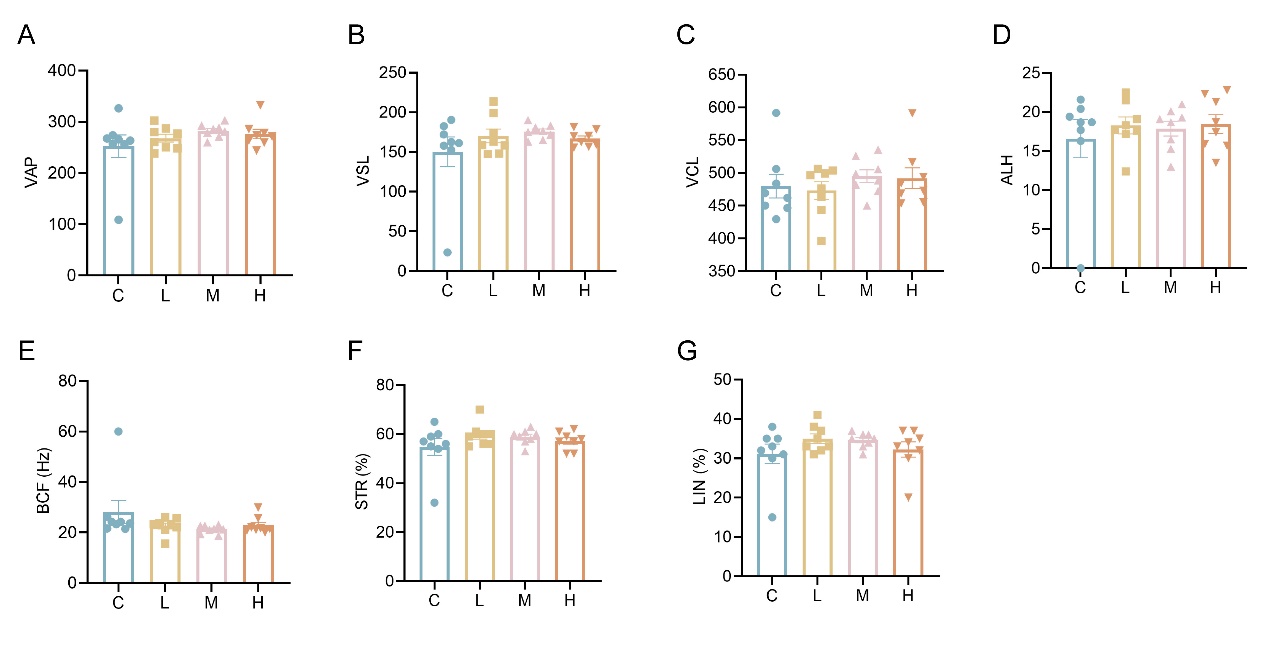
**

**Figure S1. Relationship between PFHxS exposure and sperm characteristics in mice. (A-G)** Represents VAP, VSL, VCL, ALH, BCF, STR, and LIN, respectively (n = 8).


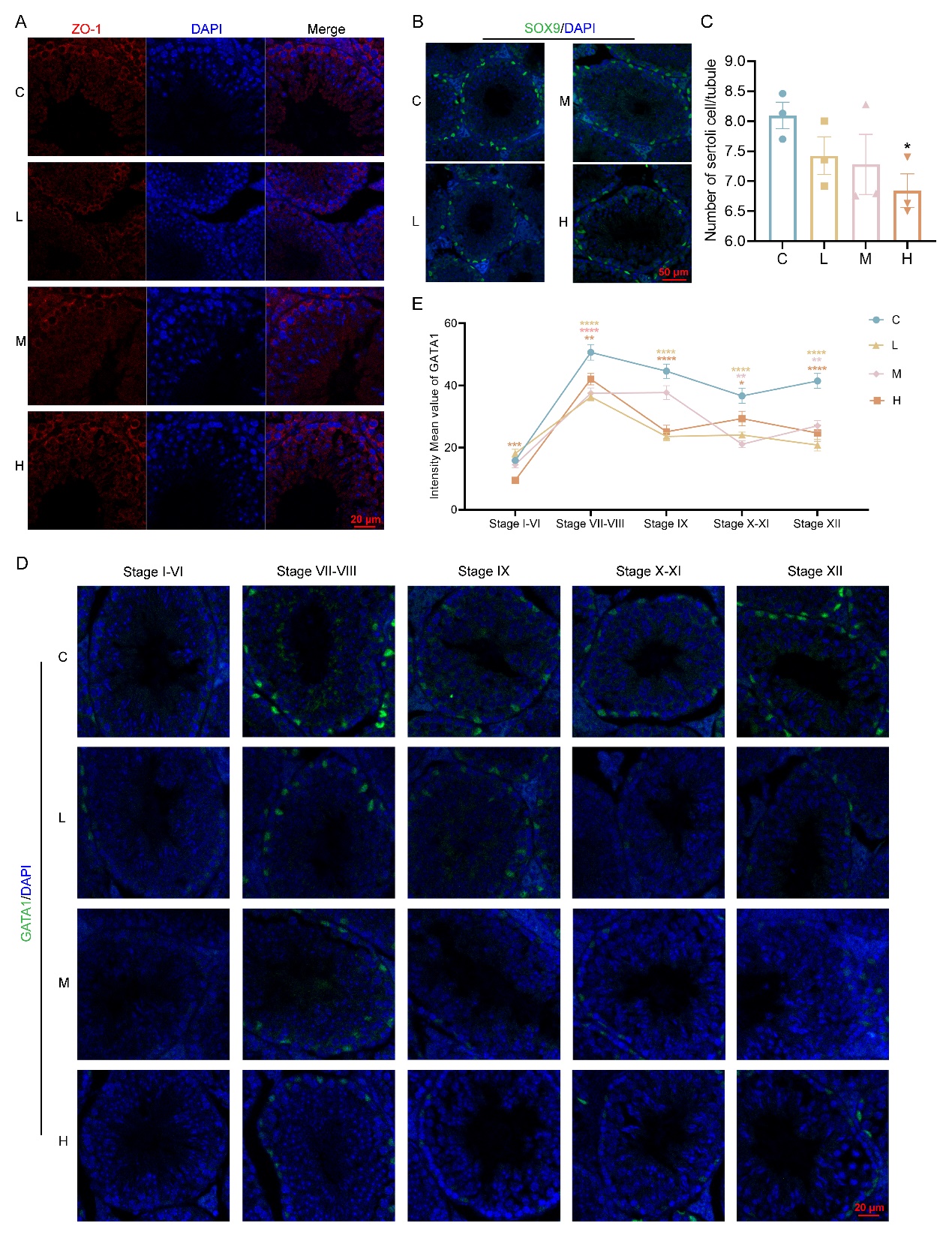


**Figure S2. Immunofluorescence analysis of the localization and expression of proteins related to BTB and Sertoli cell function. (A-E)** Immunofluorescence analysis of ZO-1 (A), SOX9 (B, C) and GATA-1 (D, E) protein expression and localization in mouse testis. Scale bar, 20 μm and 50 μm (n = 3). ^*^*P* < 0.05, ^**^ *P* < 0.01, ^***^ *P* < 0.001, and ^****^ *P* < 0.0001 represented statistically significant difference by one-way analysis.


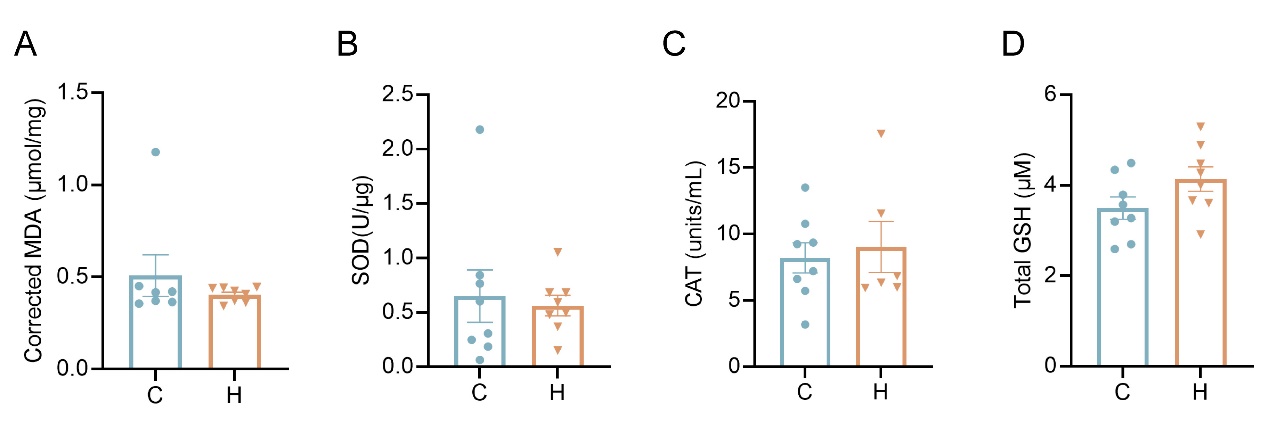


**Figure S3. Evaluation of oxidative stress and antioxidant defense. (A-D)** MDA (A), SOD (B), CAT (C), and total GSH (D) were measured in mice testes (n = 6-8).


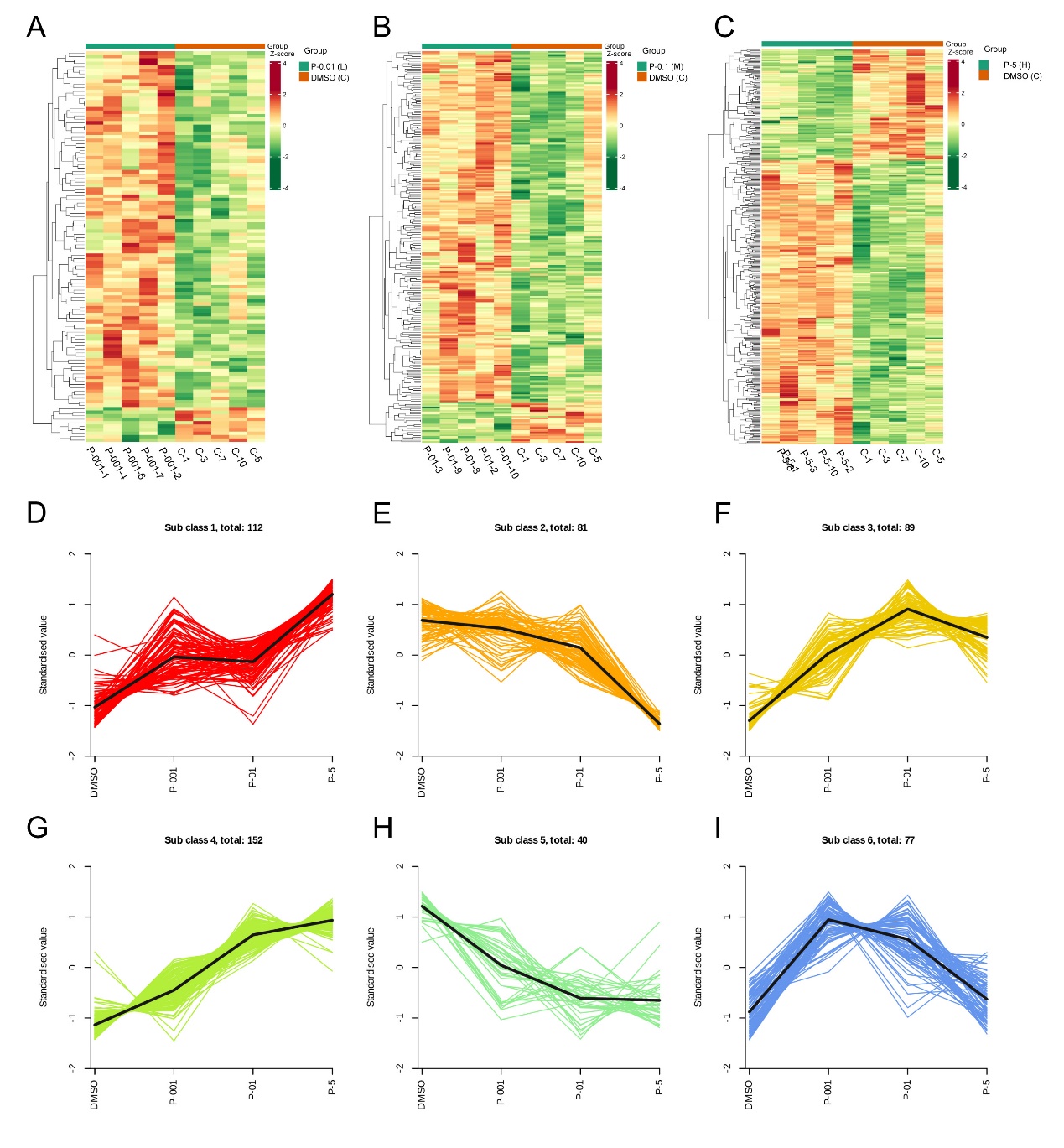


**Figure S4. Differential metabolite profiles after PFHxS-treatment in mouse testis. (A-C)** Heatmap of the differential metabolites in three compared sets (n = 5). **(D-I)** K-means cluster of differential metabolites, DMSO (C group), P-001 (L group), P-01 (M group), and P-5 (H group) (n = 5).


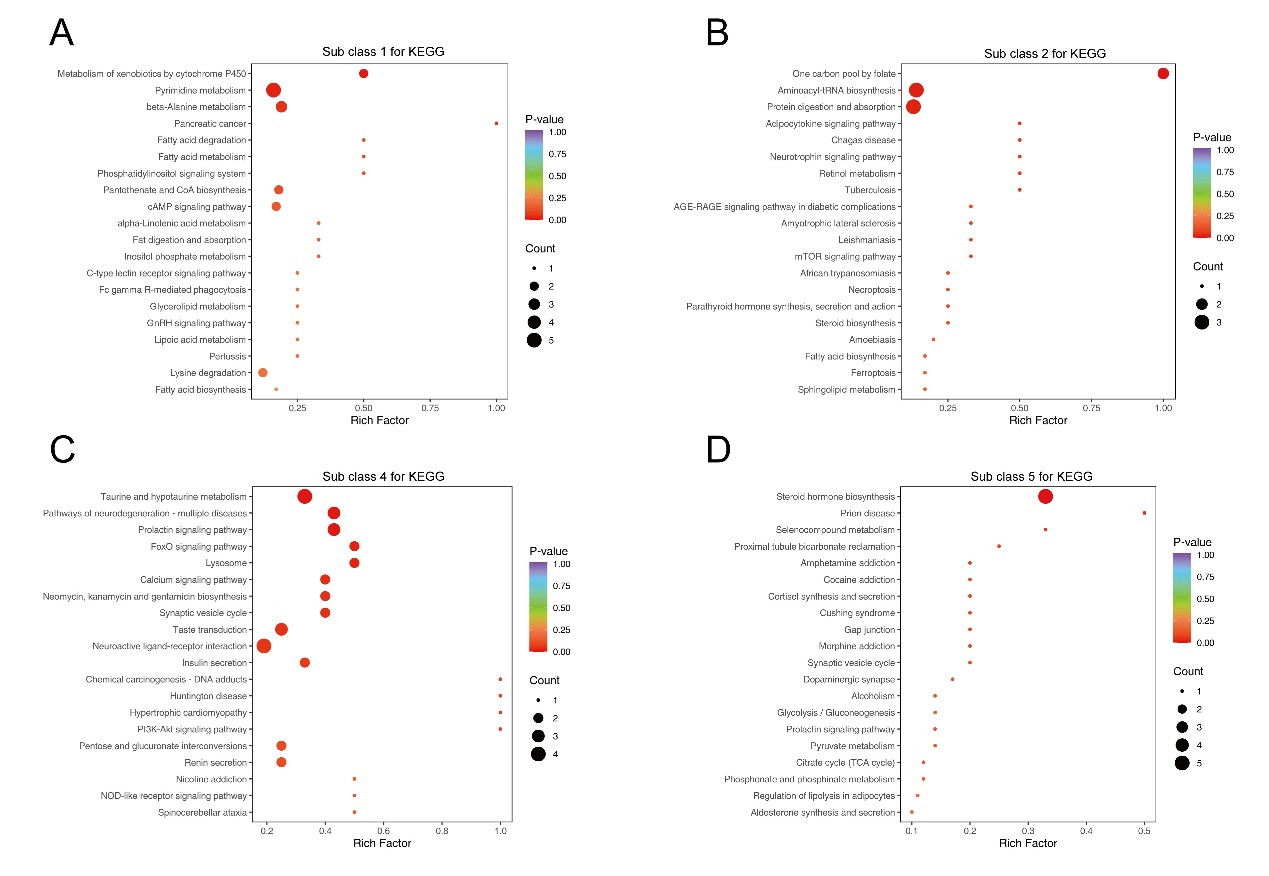


**Figure S5. KEGG analysis of subgroup differential metabolites based on K-Means cluster analysis data.** (A-D) Subclass 1, 2, 4, and 5, respectively (n = 5).
